# Knockout of a key gene of the nicotine biosynthetic pathway severely affects tobacco growth under field, but not greenhouse conditions

**DOI:** 10.1186/s13104-022-06188-9

**Published:** 2022-09-06

**Authors:** William A. Smith, Yuki Matsuba, Ralph E. Dewey

**Affiliations:** Department of Crop and Soil Sciences, North Carolina State University, Raleigh, NC USA

**Keywords:** Quinolate phosphoribosyltransferase, CRISPR/Cas9, Low nicotine, *Nicotiana tabacum*

## Abstract

**Objective:**

There is great interest in developing tobacco plants containing minimal amounts of the addictive compound nicotine. Quinolate phosphoribosyltransferase (QPT) is an important enzyme both for primary (NAD production) and secondary (pyridine alkaloid biosynthesis) metabolism in tobacco. The duplication of an ancestral *QPT* gene in *Nicotiana* species has resulted in two closely related *QPT* gene paralogs: *QPT1* which is expressed at modest levels throughout the plant, and *QPT2* which is coordinately regulated with genes dedicated to alkaloid biosynthesis. This study evaluated the utility of knocking out *QPT2* function as a means for producing low alkaloid tobacco plants.

**Results:**

CRISPR/Cas9 vectors were developed to specifically mutate the tobacco *QPT2* genes associated with alkaloid production. Greenhouse-grown *qpt2* plants accumulated dramatically less nicotine than controls, while displaying only modest growth differences. In contrast, when *qpt2* lines were transplanted to a field environment, plant growth and development was severely inhibited. Two conclusions can be inferred from this work: (1) *QPT1* gene function alone appears to be inadequate for meeting the QPT demands of the plant for primary metabolism when grown in a field environment; and (2) the complete knockout of *QPT2* function is not a viable strategy for producing agronomically useful, low nicotine tobaccos.

**Supplementary Information:**

The online version contains supplementary material available at 10.1186/s13104-022-06188-9.

## Introduction

Nicotine is the most abundant alkaloid produced in tobacco plants, typically accounting for > 90% of the total alkaloid pool, and 2–6% of the leaf biomass (dry weight) when grown commercially. In recent years there has been increased interest in developing tobacco varieties that possess very low levels of nicotine while retaining acceptable agronomic qualities. Two of the motivating factors behind this interest include: (1) evidence from numerous clinical studies that have shown that smokers who switch to cigarettes containing nicotine levels below that capable of sustaining an addiction response will smoke less and/or find it easier to quit (reviewed in [1]); and (2) the possibility that the US Food and Drug Administration (FDA) may mandate such reductions in future cigarette products [2]. From a plant genetic perspective, lowering the nicotine content of the leaf can be accomplished through the utilization of naturally occurring mutations, genetic engineering, and genome editing [3–7]. In the majority of cases reported to date, however, the genetic alterations of nicotine content do not decrease the nicotine levels below FDA’s target threshold to assure the failure to initiate or sustain an addiction response and/or are negatively associated with agronomic and quality traits [8]. Thus, there is a need to discover alternative genetic traits that can confer sufficiently low levels of nicotine while retaining agronomic acceptability.

Genes encoding the enzyme quinolate phosphoribosyltransferase (QPT) are intriguing targets for reducing nicotine in tobacco. Unlike most steps of the nicotine biosynthetic pathway that are dedicated solely to alkaloid production, QPT plays a critical role in primary metabolism as well, serving as the entry point into the pyridine nucleotide pathway responsible for production of the ubiquitous cellular co-factor NAD [9]. Characterization of the *QPT* gene family in *Nicotiana* revealed the presence of two closely-related paralogs: *QPT1* which is constitutively expressed at a relatively low level throughout the plant, and *QPT2* whose expression is induced at high levels in root tissue in response to stimuli known to activate alkaloid production [10, 11]. These distinct expression patterns led to the proposal that the ancient duplication of a housekeeping *QPT* gene resulted in the evolution of a paralog primarily dedicated to alkaloid biosynthesis (*QPT2*) while retaining a copy that could serve the needs for primary metabolism (*QPT1*) [10].

Because *QPT1* and *QPT2* share 94% nucleotide sequence identity, it would be difficult, if not impossible, to use techniques such as RNA interference (RNAi) or antisense-suppression to down regulate one paralog without simultaneously inhibiting the other. Indeed, when *QPT* gene function was suppressed using an RNAi construct driven by the CaMV 35S promoter, total *QPT* transcript reduction lead to severe stunting, abnormal leaf and flower morphologies, and photosynthetic deficiencies in lab and greenhouse grown plants [12]. When an anti-sense suppression strategy was used to down regulate *QPT* activity specifically within the root, however, low nicotine lines that were otherwise phenotypically normal were reported [3]. Because neither of these strategies would be predicted to uniquely repress a single *QPT* paralog, they could not address the issue of whether the targeted disruption of *QPT2* alone represents a viable means for generating low nicotine tobaccos. In this study, genome editing was used to introduce frame-shift mutations in the *QPT2* genes of two commercial tobacco varieties, and alkaloid profiles and growth characteristics were measured in plants grown in both a greenhouse and field environment.

## Main text

### Methods

#### Targeted mutagenesis of QPT2

The *QPT2-*specific sequence 5′-AGCCACCAAGAATACAAGAG-3′ was targeted by cloning complementary, annealed oligonucleotides into the *Bsa*I-digested sgRNA cassette of the CRISPR/Cas9 vector pRGEB31 (Addgene) as previously described [13]. A map of pRGEB31 can be found at www.addgene.org/51295/. Off-target analysis was conducted using Cas-OFFinder (http://www.rgenome.net/cas-offinder/) as described [14]. The *QPT2*-targeting vector was transformed into tobacco varieties K326 and TN90 using *Agrobacterium* as previously described [15]. T_0_ plants were screened for mutations in *QPT2_T*, *QPT2_S, QPT1_T* and *QPT1_S* by PCR amplification using primers specific for each gene that flank the target site, followed by DNA sequence analysis by Sanger sequencing, using the forward or reverse primers as sequencing primers. The presence of CRISPR/Cas-induced indels was determined by direct examination of the sequencing chromatograms. The primers used in this study and PCR conditions are listed in Additional file 1: Table S1. DNA sequencing was conducted at the NCSU Genomic Sciences Laboratory (https://research.ncsu.edu/gsl). T_1_ generation lines were screened for the absence of the *hptII* selectable marker, as well as the absence of segregating WT *QPT2* loci. GenBank accession numbers for the *QPT* gene family are: XM_016656561 (*QPT1_T*), XM_016652759 (*QPT1_S*), NM_001326216 (*QPT2_T*) and NM_001326058 (*QPT2_S*).

#### Greenhouse and field growth and evaluation

Seeds were germinated and grown in a growth chamber for 48 days. Twelve plants per genotype (T_2_ generation) were subsequently transplanted to 9″ pots and transferred to a greenhouse. Each plant was topped upon the first appearance of bud formation and suckers were removed manually for the next 10 days. The mid-rib was removed from leaves selected for alkaloid analysis and the lamina was dried to completeness in a drying oven. Alkaloid analysis was conducted by the NCSU Tobacco Analytical Services Lab as described previously [16]. For field-grown plants, seeds were sown on float trays in a greenhouse for 73 days with occasional mowing to promote root growth. Transplants were transferred to the field and grown using standard agronomic production practices. Statistical analysis was performed on the various measurements by conducting individual t-tests between mutant *qpt2* lines and their relevant WT controls.

### Results and discussion

**Fig. 1:**
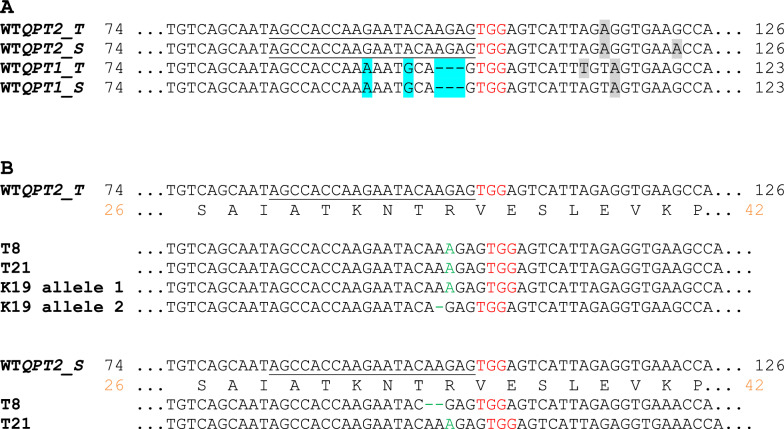
CRISPR/Cas9-induced knockout mutations in *QPT2_T* and *QPT2_S.* The 20 bp sequence targeted by the nuclease is underlined and the PAM site is shown in red type. **A** Alignment of tobacco *QPT2* and *QPT1* genes in the region of the target site. The 3 bp deletion and two single nucleotide polymorphisms that are found within the comparable 20 bp region of *QPT1_T* and *QPT1_S* are highlighted in blue. Other polymorphisms among the *QPT* genes are highlighted in gray. Gene mutations found in the three lines that were characterized in the greenhouse and field are shown in **B**. Inserted nucleotides and the positions of deleted nucleotides (dashes) are represented in green type. The numbers in black type correspond to the *QPT* cDNA sequences beginning at the start ATG codon; orange numbers represent amino acid position

To determine the effects of knocking out *QPT2* function into tobacco, two varieties representing each of the major tobacco market types, flue-cured (K326) and burley (TN90), were selected as the recipient backgrounds. Previous tobacco genome analyses revealed that TN90 possesses the *QPT2_T* and *QPT2_S* genes derived from the ancestral species *N. tomentosiformis* and *N. sylvestris,* respectively, while K326 only contains the *QPT2_T* copy [17]. A 20 bp CRISPR/Cas9 target (plus 3 bp PAM) site was selected based on the following criteria: (1) its location in near 5’ end of the gene; (2) the presence of several polymorphisms in the comparable region of *QPT1*; and (3) the absence of any other sequences the public tobacco reference genomes that matched this sequence. The target sequence shown in Fig. 1A met all these requirements. A list of all sequences in the tobacco genome that possess up to three mismatches in comparison to the *QPT2* target site as determined by the algorithm Cas-OFFinder is shown in Additional file 2: Table S2.

K326 and TN90 were transformed with the *QPT2*-targeting CRISPR/Cas9 construct and T_0_ individuals were screened for *QPT2* mutations. In K326, 15 T_0_ transformants were examined for mutations in *QTP2*; 9 possessed an indel in at least one allele of *QPT2_T* (60% efficiency). In TN90, 24 T_0_ plants were genotyped for *QPT2* mutations; 18 contained a mutation in at least one allele of *QPT2_S* (75%), and 12 contained an indel in at least one allele of *QPT2_T* (50%). One K326 plant (K19) and two TN90 plants (T8 and T21) were selected for further analysis. K19 was biallelic for a 1 bp insertion and 1 bp deletion at the *QPT2_T* locus (Fig. 1B). T8 was homozygous for the same 1 bp insertion at *QPT2_T* and was monoallelic for a 2 bp deletion at *QPT2_S*. T21 was monoallelic for a 1 bp insertion at *QPT2_T* and was homozygous for a 1 bp insertion in *QPT2_S*. Each of these mutations caused frame shifts that would lead to premature stop codons anywhere from 12 to 76 bp downstream of the mutation. Given that only the first 33 aa of what is normally a 351 aa protein would be retained in each of these mutant loci, it was assumed that these mutations would result in the complete loss of gene function. PCR amplification and sequence analysis of the *QPT1* genes in these same individuals confirmed that no *QPT1* gene had been mutated. Plants K19, T8 and T21 were self-pollinated and numerous T_1_ progeny were genotyped to identify those that had lost the CRISPR/Cas9 vector and were homozygous mutant at all *QPT2* loci. Chromatograms of each *QPT* gene in the region targeted for mutagenesis in T_1_ generation plants of lines K19, T8 and T21 are shown in Additional file 3: Fig. S1.

Twelve T_2_ generation individuals for each of the *qpt2* mutant genotypes, along with their corresponding WT controls, were randomized within the same greenhouse and grown to maturity. Upon the first observation of bud formation, the date was recorded and the plant was topped by excising the floral meristem together with the first 6–8 immature leaves. The plants were grown an additional 10 days post-topping, at which time the following data were collected: plant height, leaf number and total leaf weight. In addition, an equivalently positioned leaf (5th or 6th leaf from the top) was selected for alkaloid analysis.

**Fig. 2:**
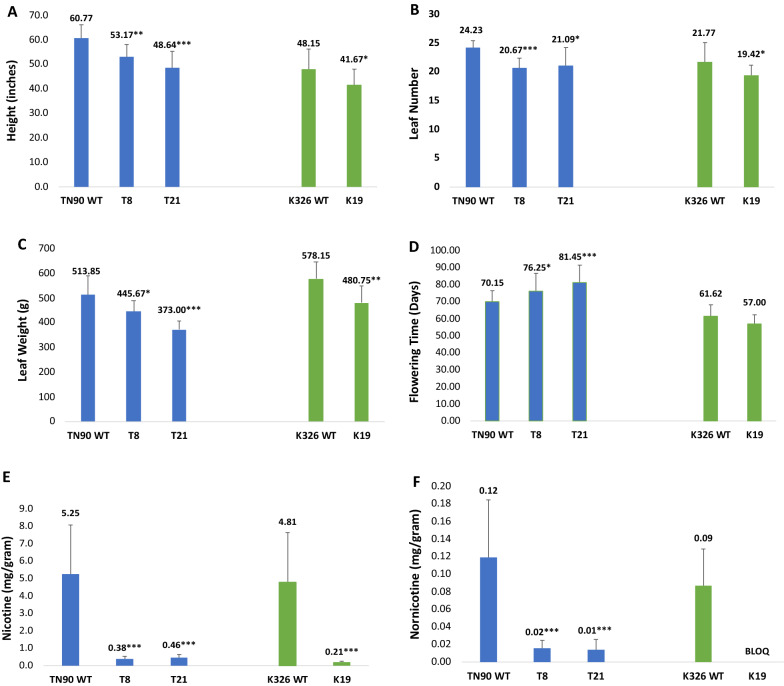
Growth characteristics and alkaloid content of greenhouse-grown tobacco plants possessing knockout mutations in *QPT2* genes. Blue bar graphs show data comparisons among mutant lines T8 and T21 and their corresponding TN90 WT control; green bar graphs depict the K19 mutant and its K326 WT control. Measurements include: plant height (**A**), leaf number (**B**), leaf weight (**C**), flowering time (**D**), nicotine (**E**) and nornicotine (**F**). For flowering time, days = days post-transplant and transfer to the greenhouse. Means and standard error bars are shown above each genotype. Asterisks indicate significant differences between *qpt2* mutant lines and their corresponding WT control at P < 0.05 (*), P < 0.01 (**) and P < 0.001 (***) as determined by t-tests. BLOQ, below level of quantification

In both the TN90 and K326 backgrounds, plant height was reduced between 13 and 20%, and leaf number decreased by an average of 2–4 leaves per plant in the *qpt2* mutant lines (Fig. 2A and B). Total leaf weight was decreased approximately 13% and 17% in lines T8 and K19, respectively, in comparison to their WT controls; a more substantial decrease of 27% was observed in line T21 (Fig. 2C). Line T21 also displayed the greatest difference in flowering time, with buds appearing on average 11 days later than WT TN90 plants. T8 plants flowered an average of 6 days later than WT (Fig. 2D). Flue-cured line K19 flowered on average 4.5 days earlier than its WT control, but this difference was not considered statistically significant.

In contrast to the modest differences in overall growth phenotypes observed between WT tobaccos and their corresponding *qpt2* mutants, alkaloid profiles differed dramatically (Fig. 2E and F). Nicotine levels in the *qpt2* lines were reduced between 91 and 96%; similar reductions were observed in nornicotine content. Anatabine and anabasine comparisons were not included because their levels were below the level of quantification in the majority of the *qpt2* individuals.

**Fig. 3:**
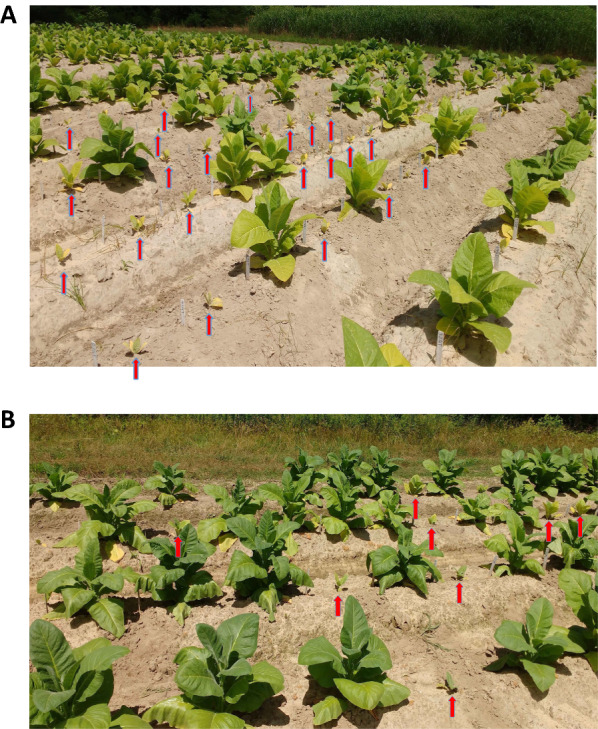
Tobacco plants harboring *qpt2* knockout mutations display a severe growth phenotype when grown in a field environment. **A** Control and mutant lines T8 and T21 in the TN90 background; **B** Controls and mutant line K19 in the K326 background. Tester and control lines were randomized within rows. Red arrows point to plants homozygous for *qpt2* mutations; full-sized plants correspond to control and other entries. Picture was taken 32 days after transplant

Overall, the results from the greenhouse study suggested that knocking out *QPT2* function in tobacco may represent a viable means for producing low alkaloid tobaccos, and warranted further evaluation in a field environment. In keeping with traditional agronomic practice, seeds from each line were initially planted in float trays in a greenhouse prior to transplanting the young plantlets to the field. Within the greenhouse float trays there were no obvious phenotypic differences between the *qpt2* mutant and control lines (W. Smith, personal observation). Tobaccos in the burley (T8, T21 and TN90 WT) and flue-cured (K19, K326 WT and an unrelated low nicotine line in K326) backgrounds were transplanted to the field in separate experimental plots, comprised of 30 plants per line planted in a randomized complete block design. Surprisingly, the growth of all three *qpt2* lines was extraordinarily suppressed in the field. By 32 days post-transplant, *qpt2* individuals were just marginally larger than when transplanted from the float trays (Fig. 3). As the growing season continued and the control plants grew large, shading provided an additional impediment to their growth. As a result, none of the *qpt2* lines within the designed experiments grew taller than 30 cm, nor did they flower, which precluded the ability to obtain meaningful alkaloid data. By chance, however, extra K19 plants had been chosen to serve as border rows for an unrelated experiment within the same field. Despite remaining stunted throughout the entire growth season, in the absence of undue shading competition, most of the K19 border plants developed to the extent where they initiated flowering. The K19 border plants were topped, treated with suckercide and a subset assayed for alkaloid content. Additional file 4: Fig. S2 shows representative K19 border plants on the day of harvest, and how the average nicotine content of the *qpt2* plants was 99% reduced in comparison to that observed in K326 WT plants grown in a different part of the same field.

Our results support the notion that *QPT1* genes alone can largely accommodate the plant’s need for NAD, and other products of the pyridine nucleotide cycle, under conditions of minimal environmental stress, and that an additional contribution from *QPT2* genes is required when grown in the field. The physiological demands of outdoor growth that necessitate this additional contribution are unknown, but may include: (1) stresses associated with transplant shock when transferred from the greenhouse to the field; (2) mechanical stresses caused by exposure to wind; and (3) increased temperature extremes and variable water availability. Although genome editing-mediated knockout of *QPT2* loci yielded tobaccos with dramatically reduced leaf nicotine content, the associated negative impacts on plant growth and development under standard field conditions precludes the use of lines possessing these mutations for the commercial production of low nicotine tobaccos.

### Limitations

The main limitation was that this study was conducted during a single year in a single field environment. It is thus possible that the detrimental effects of knocking out *qpt2* function may not always be as extreme as documented in this report. Nevertheless, the fact that field growth can, if even only under certain environments, result in the type of severe growth reduction reported here would prevent consideration of mutations of this nature for commercial deployment.

## Supplementary Information

**Additional file 1: Table S1. **Primers and PCR conditions used in this study.

**Additional file 2: Table S2. **Sequences most closely related to *QPT2 *target sequence in the TN90 reference genome as determined by Cas-OFFinder.

**Additional file 3: Figure S1. **Chromatograms of the tobacco *QPT* genes in WT (**A**) and genome edited backgrounds T8 (**B**), T21 (**C**) and K19 (**D**). To help align the chromatogram information with the sequences shown in Fig. 1, the ‘TGG’ PAM sites are indicated. Sequences for *QPT2_T, QPT2_S* and *QPT1_T* are shown in the forward direction; *QPT1_S* sequences are shown in the reverse complement. For line K19 plants that are heterozygous for the alternative mutant alleles in *QPT2_T*, each allele can be read independently from the chromatogram after the point where the two patterns diverge as shown in (**D**).

**Additional file 4: Figure S2**. K19 (*qpt2_t/qpt2_t*) border row plants on the day of field harvest (110 days after transplant). Average nicotine content from 19 topped K19 border plants (K326 qpt2qpt2) and 19 topped K326 WT plants grown in a separate part of the field is shown on the accompanying graph. Means ± standard errors of means are shown. The difference in nicotine content was significant at P < 0.001 as determined by a t-test.

## Data Availability

The datasets used and/or analyzed during the current study are available from the corresponding author on reasonable request.

## Abbreviations

QPT: Quinolate phosphoribosyltransferase
NAD: Nicotinamide adenine dinucleotide
CRISPR/Cas9: Clustered regularly interspaced short palindromic repeat/CRISPR-associated 9
RNAi: RNA interference
CaMV: Cauliflower mosaic virus
PCR: Polymerase chain reaction
bp: Base pairs
aa: Amino acids
WT: Wild type
sgRNA: Single-guide RNA
PAM: Protospacer adjacent motif
BLOQ: Below level of quantification
