# Supplementary material for: Knockout of a key gene of the nicotine biosynthetic pathway severely affects tobacco growth under field, but not greenhouse conditions
Source: BMC Res Notes. 2022 Sep 6;15:291. doi: 10.1186/s13104-022-06188-9 (PMC9450462; doi:10.1186/s13104-022-06188-9)
Supplement: Supplementary file 1 — Additional file 1: Table S1. Primers and PCR conditions used in this study. [file 13104_2022_6188_MOESM1_ESM.docx]

**Additional file 1: Table S1. Primers and PCR conditions used in this study**

| Gene | Forward primer 5’-3’ | Reverse primer 5’-3’ | Denaturation  Temp/time | Annealing  Temp/time | Extension  Temp/time |
| --- | --- | --- | --- | --- | --- |
| *QPT2_T* | ATAAACCATGTTTAGGGCTC | TGTTACTATGCACTAACAGT | 98^o^C/10 sec | 60^o^C/15 sec | 72^o^C/15sec |
| *QPT2_S* | AAAACCATGTTTAGAGCTA | AAGTTCTATGCACTAACGGG | 98^o^C/10 sec | 60^o^C/15 sec | 72^o^C/15sec |
| *QPT1_T* | CTGCAATAGTCCACCCTAATGC | ACTTCCTAGCGCGAATCTGG | 98^o^C/10 sec | 64^o^C/15 sec | 72^o^C/15sec |
| *QPT1_S* | TAAATTTTGTCTGTAAATTGCAGG | ATCCCCAGCATCTTCAGAGAG | 98^o^C/10 sec | 60^o^C/15 sec | 72^o^C/15sec |
| *hptII* | GTGTACGCCCGACAGTCCCGGC | CCCGATTCCGGAAGTGCTTGAC | 98^o^C/10 sec | 63^o^C/10 sec | 72^o^C/15sec |

All reactions were initiated with a 30 sec denaturation at 98^o^C for 30 sec, and terminated with a 7 min extension at 72^o^C after 33 cycles. Reactions were conducted using Phusion Taq and reaction buffer (New England Biolabs – cat. #M0530L).
