# Supplementary material for: Knockout of a key gene of the nicotine biosynthetic pathway severely affects tobacco growth under field, but not greenhouse conditions
Source: BMC Res Notes. 2022 Sep 6;15:291. doi: 10.1186/s13104-022-06188-9 (PMC9450462; doi:10.1186/s13104-022-06188-9)
Supplement: Supplementary file 2 — Additional file 2: Table S2. Sequences most closely related to QPT2 target sequence in the TN90 reference genome as determined by Cas-OFFinder. [file 13104_2022_6188_MOESM2_ESM.docx]

**Additional file 2: Table S2. Sequences most closely related to *QPT2* target sequence in the TN90 reference genome as determined by Cas-OFFinder.** Nucleotides that are polymorphic to the *QPT2* target site are in lower case type and shaded green. PAM site is shown in red type.

| Target^a^ | Contigs | Position^b^ | Mismatches | Predicted Gene |
| --- | --- | --- | --- | --- |
| AGCCACCAAGAATACAAGAGNGG  AGCCACCAAGAATACAAGAGTGG | AYMY01004730 | 24599 (+) | 0 | *QPT2_T* |
| AGCCACCAAGAATACAAGAGNGG  AGCCACCAAGAATACAAGAGTGG | AYMY01258031 | 540 (-) | 0 | *QPT2_S* |
| AGCCACCAAGAATACAAGAGNGG  AGCCACaAgGAATACAAGAGTGG | AYMY01155088 | 38193 (-) | 2 | In a predicted intron of a predicted NADH dehydrogenase iron-sulfur protein 4 |
| AGCCACCAAGAATACAAGAGNGG  AGgtACCAAGAATACAAGtGTGG | AYMY01211211 | 43837 (+) | 3 | Serrate RNA effector molecule-like |
| AGCCACCAAGAATACAAGAGNGG  AGaCAaCAAGAAaACAAGAGGGG | AYMY01417781 | 142 (-) | 3 | No matches to a predicted gene/protein |
| AGCCACCAAGAATACAAGAGNGG  AGaCAaCAAGAAaACAAGAGGGG | AYMY01337181 | 348 (-) | 3 | No matches to a predicted gene/protein |
| AGCCACCAAGAATACAAGAGNGG  tGCCAaCAAGAATACAAGAaAGG | AYMY01141499 | 40970 (-) | 3 | No matches to a predicted gene/protein |
| AGCCACCAAGAATACAAGAGNGG  AGCCtCtAAGAATACAAaAGAGG | AYMY01149167 | 295 (-) | 3 | No matches to a predicted gene/protein |
| AGCCACCAAGAATACAAGAGNGG  AGgtACCAAGAATACAAGtGTGG | AYMY01008458 | 55219 (+) | 3 | Serrate RNA effector molecule-like |
| AGCCACCAAGAATACAAGAGNGG  AGaCAaCAAGAAaACAAGAGGGG | AYMY01326227 | 398 (-) | 3 | No matches to a predicted gene/protein |
| AGCCACCAAGAATACAAGAGNGG  AGaCAaCAAGAAaACAAGAGGGG | AYMY01273769 | 298 (-) | 3 | No matches to a predicted gene/protein |
| AGCCACCAAGAATACAAGAGNGG  AGaCAaCAAGAAaACAAGAGGGG | AYMY01265104 | 338 (-) | 3 | No matches to a predicted gene/protein |
| AGCCACCAAGAATACAAGAGNGG  AGaCAaCAAGAAaACAAGAGGGG | AYMY01236249 | 158 (-) | 3 | No matches to a predicted gene/protein |
| AGCCACCAAGAATACAAGAGNGG  AGaCAaCAAGAAaACAAGAGGGG | AYMY01102458 | 122 (-) | 3 | No matches to a predicted gene/protein |
| AGCCACCAAGAATACAAGAGNGG  AGaCAaCAAGAAaACAAGAGGGG | AYMY01058827 | 835 (-) | 3 | No matches to a predicted gene/protein |
| AGCCACCAAGAATACAAGAGNGG  AGaCAaCAAGAAaACAAGAGGGG | AYMY01373040 | 285 (+) | 3 | No matches to a predicted gene/protein |
| AGCCACCAAGAATACAAGAGNGG  AGaCAaCAAGAAaACAAGAGGGG | AYMY01372890 | 155 (+) | 3 | No matches to a predicted gene/protein |
| AGCCACCAAGAATACAAGAGNGG  AGaCAaCAAGAAaACAAGAGGGG | AYMY01325179 | 38 (+) | 3 | No matches to a predicted gene/protein |
| AGCCACCAAGAATACAAGAGNGG  AGaCAaCAAGAAaACAAGAGGGG | AYMY01213953 | 3 (+) | 3 | No matches to a predicted gene/protein |
| AGCCACCAAGAATACAAGAGNGG  AGaCAaCAAGAAaACAAGAGGGG | AYMY01213134 | 182 (+) | 3 | No matches to a predicted gene/protein |

^a^For all alignments, the sgRNA recognition sequence is shown on the top.

^b^A “+” after the number indicates that the 23 bp target, or off-target, is found in the same orientation as the GenBank accession; a “-“ means that it is found in the reverse complement orientation.
