# Supplementary material for: Knockout of a key gene of the nicotine biosynthetic pathway severely affects tobacco growth under field, but not greenhouse conditions
Source: BMC Res Notes. 2022 Sep 6;15:291. doi: 10.1186/s13104-022-06188-9 (PMC9450462; doi:10.1186/s13104-022-06188-9)
Supplement: Supplementary file 3 — Additional file 3: Figure S1. Chromatograms of the tobacco QPT genes in WT (A) and genome edited backgrounds T8 (B), T21 (C) and K19 (D). To help align the chromatogram information with the sequences shown in Fig. 1, the ‘TGG’ PAM sites are indicated. Sequences for QPT2_T, QPT2_S and QPT1_T are shown in the forward direction; QPT1_S sequences are shown in the reverse complement. For line K19 plants that are heterozygous for the alternative mutant alleles in QPT2_T, each allele can be read independently from the chromatogram after the point where the two patterns diverge as shown in (D). [file 13104_2022_6188_MOESM3_ESM.docx]

1. **WT chromatograms**

QPT2_T target seq _


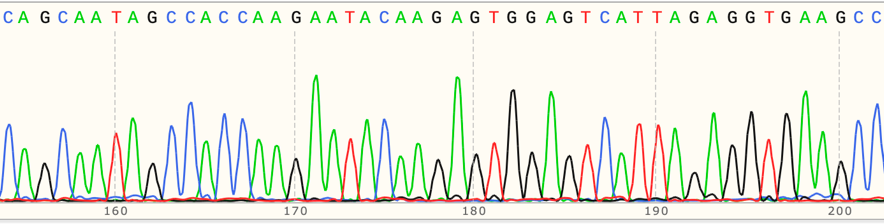

QPT2_S target seq _


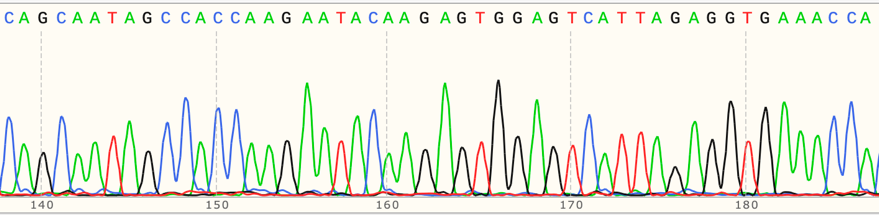

QPT1_T multiple polymorphs _


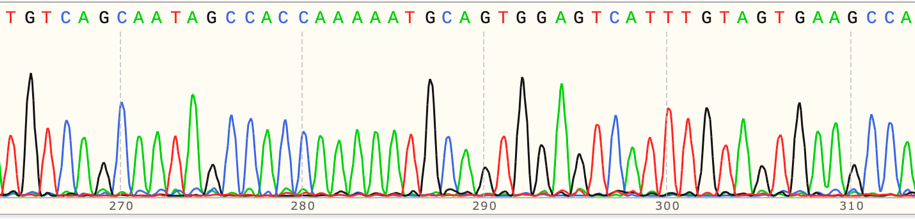

QPT1_S multiple polymorphs _


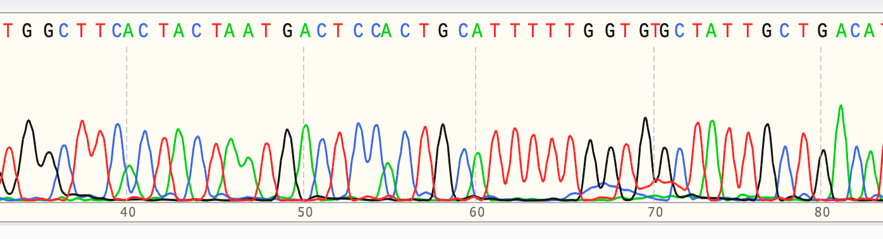


1. **T8 chromatograms**

QPT2_T target seq _ 1 bp insertion


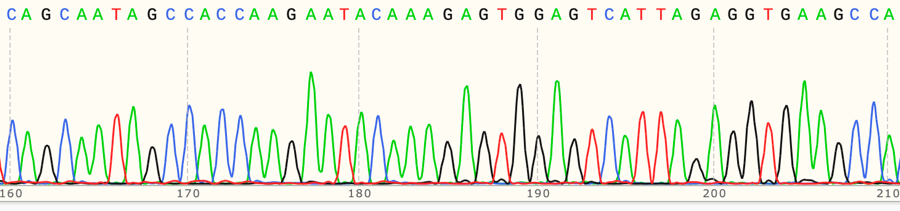

QPT2_S target seq _ 2 bp deletion


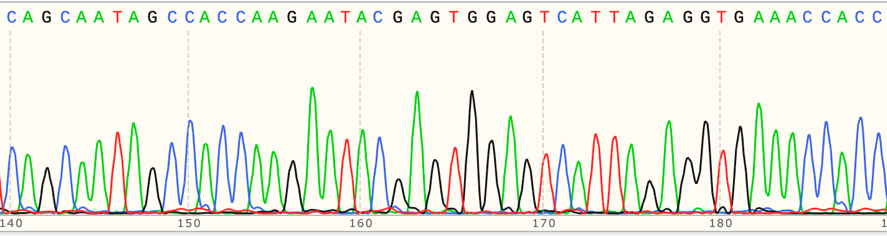

QPT1_T (WT)


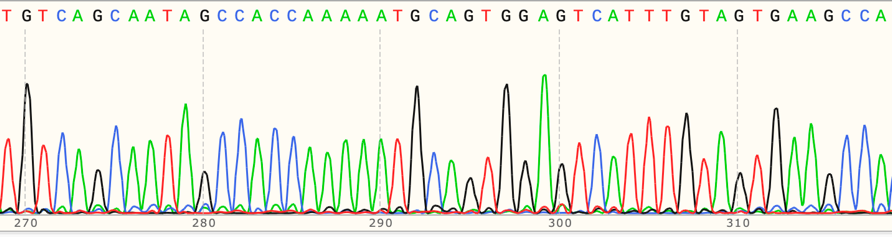

QPT1_S (WT)


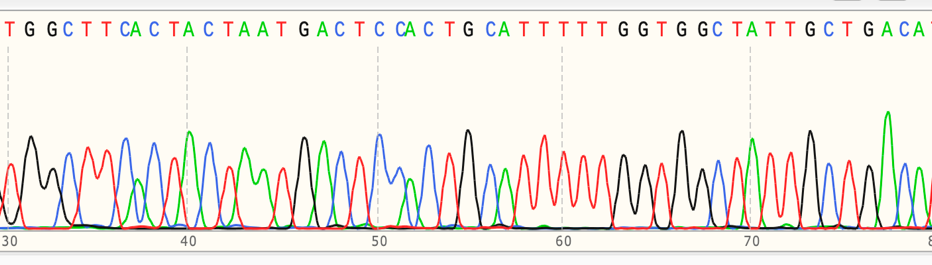


1. **T21 chromatograms**

QPT2_T target seq _ 1 bp insertion


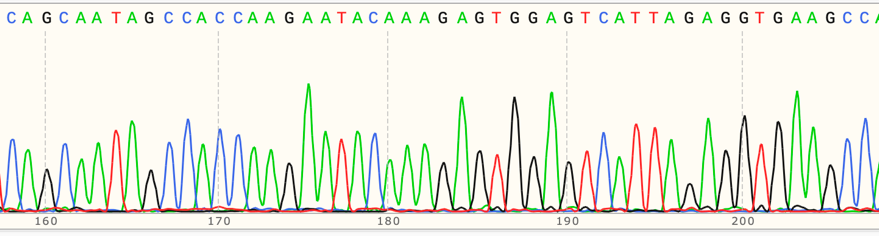

QPT2_S target seq _ 1 bp insertion


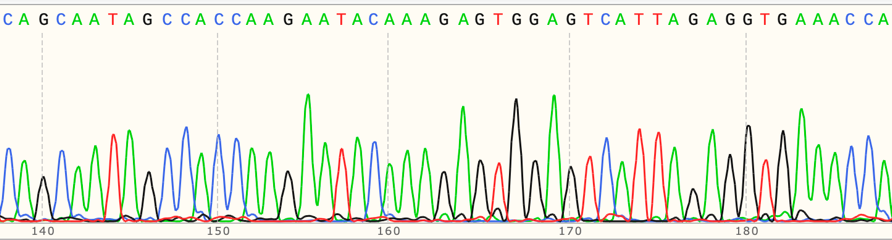

QPT1_T (WT)


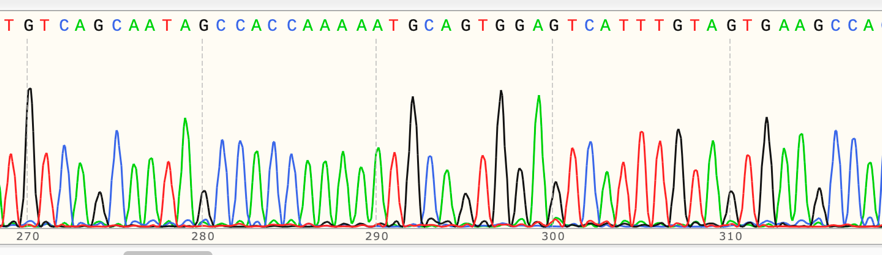

QPT1_S (WT)


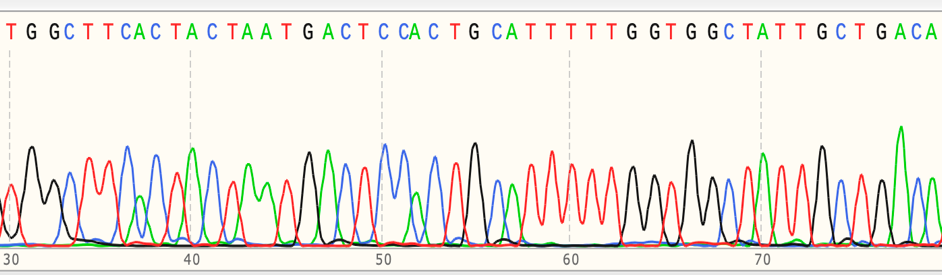


1. **K19 Chromatograms**

QPT2_T (heterozygous for 1 bp deletion / 1bp insertion)

Allele 1 (1 bp ins)


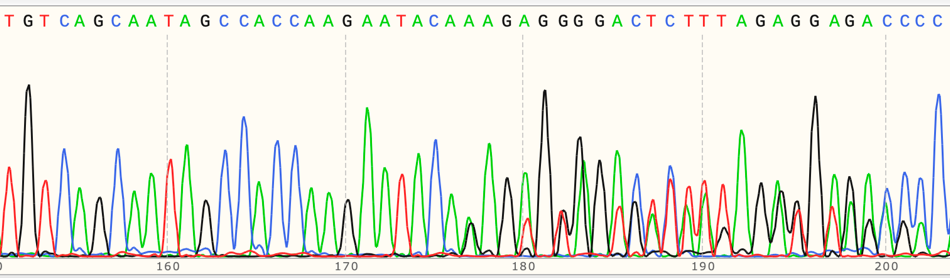


Allele 2 (1 bp del)

QTP1_T (WT)


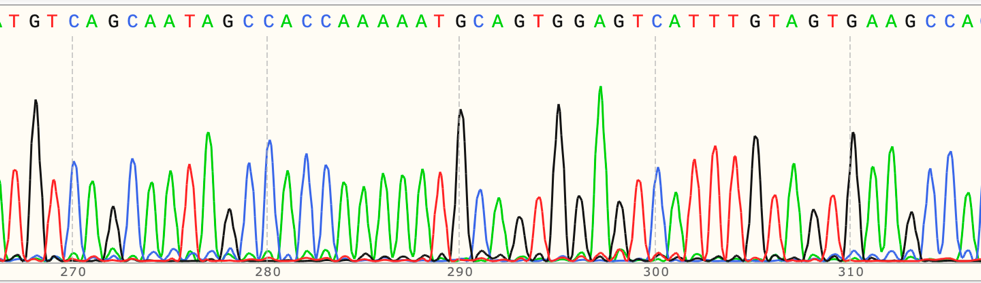


**Additional file 3: Figure S1.** Chromatograms of the tobacco *QPT* genes in WT (**A**) and genome edited backgrounds T8 (**B**), T21 (**C**) and K19 (**D**). To help align the chromatogram information with the sequences shown in Fig. 1, the ‘TGG’ PAM sites are indicated. Sequences for *QPT2_T, QPT2_S* and *QPT1_T* are shown in the forward direction; *QPT1_S* sequences are shown in the reverse complement. For line K19 plants that are heterozygous for the alternative mutant alleles in *QPT2_T*, each allele can be read independently from the chromatogram after the point where the two patterns diverge as shown in (**D**).
