# Supplementary material for: Knockout of a key gene of the nicotine biosynthetic pathway severely affects tobacco growth under field, but not greenhouse conditions
Source: BMC Res Notes. 2022 Sep 6;15:291. doi: 10.1186/s13104-022-06188-9 (PMC9450462; doi:10.1186/s13104-022-06188-9)
Supplement: Supplementary file 4 — Additional file 4: Figure S2. K19 (qpt2_t/qpt2_t) border row plants on the day of field harvest (110 days after transplant). Average nicotine content from 19 topped K19 border plants (K326 qpt2qpt2) and 19 topped K326 WT plants grown in a separate part of the field is shown on the accompanying graph. Means ± standard errors of means are shown. The difference in nicotine content was significant at P < 0.001 as determined by a t-test. [file 13104_2022_6188_MOESM4_ESM.pptx]

## Slide 1
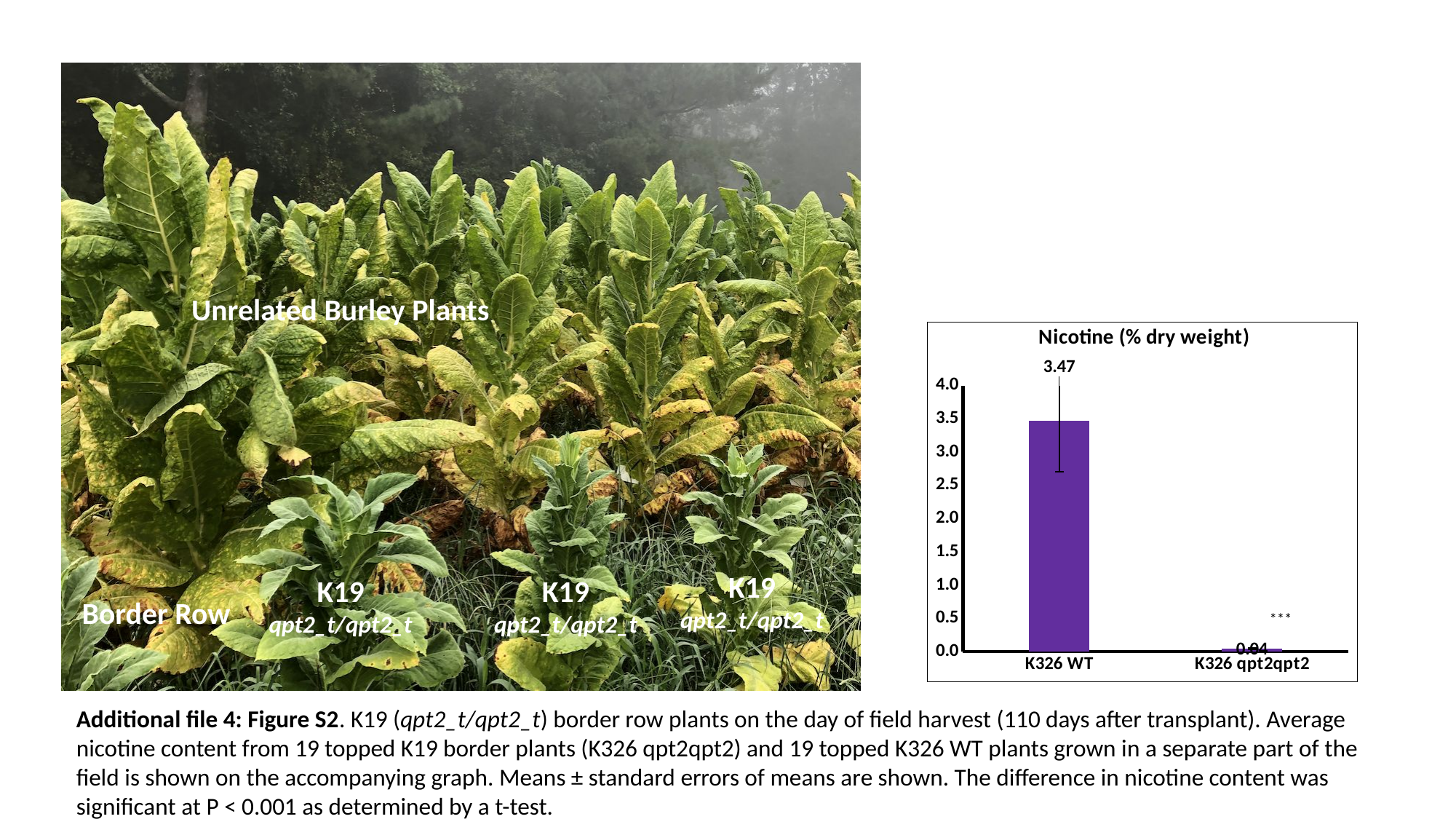

Unrelated Burley Plants
### Chart: Nicotine (% dry weight)
| Category | |
|---|---|
| K326 WT | 3.4730210526315792 |
| K326 qpt2qpt2 | 0.04314736842105262 |K19
qpt2_t/qpt2_t
K19
qpt2_t/qpt2_t
K19
qpt2_t/qpt2_t
Border Row
***
Additional file 4: Figure S2. K19 (qpt2_t/qpt2_t) border row plants on the day of field harvest (110 days after transplant). Average nicotine content from 19 topped K19 border plants (K326 qpt2qpt2) and 19 topped K326 WT plants grown in a separate part of the field is shown on the accompanying graph. Means ± standard errors of means are shown. The difference in nicotine content was significant at P < 0.001 as determined by a t-test.
